# Boundary-dependent mechanical properties of graphene annular under in-plane circular shearing via atomistic simulations

**DOI:** 10.1038/srep41767

**Published:** 2017-02-13

**Authors:** Yinfeng Li, Qianling Lin, Daxiang Cui

**Affiliations:** 1 Department of Engineering Mechanics, School of Naval Architecture, Ocean and Civil Engineering (State Key Laboratory of Ocean Engineering, Collaborative Innovation Center for Advanced Ship and Deep-Sea Exploration), Shanghai Jiao Tong University, Shanghai 200240, China; 2 Institute of Nano Biomedicine and Engineering, Key Laboratory for Thin Film and Microfabrication Technology of the Ministry of Education, Department of Instrument Science and Engineering, School of Electronic Information and Electrical Engineering, Shanghai Jiao Tong University, Shanghai 200240, China

## Abstract

Graphene annulus possesses special wrinkling phenomenon with wide range of potential applications. Using molecular dynamics simulation, this study concerns the effect of boundary on the mechanical properties of circular and elliptical graphene annuli under circular shearing at inner edge. Both the wrinkle characteristic and torque capacity of annular graphene can be effectively tuned by outer boundary radius and aspect ratio. For circular annulus with fixed inner radius, the critical angle of rotation can be increased by several times without sacrificing its torque capacity by increasing outer boundary radius. The wrinkle characteristic of graphene annulus with elliptical outer boundary differs markedly with that of circular annulus. Torque capacity anomalously decreases with the increase of aspect ratio, and a coupled effect of the boundary aspect ratio and the ratio of minor axis to inner radius on wrinkling are revealed. By studying the stress distribution and wrinkle characteristics, we find the decay of torque capacity is the result of circular stress concentration around the minor axis, while the nonuniform stress distribution is anomalously caused by the change of wrinkle profiles near the major axis. The specific mechanism of out-of-plane deformation on in-plane strength provides a straightforward means to develop novel graphene-based devices.

With the rapid advancement of nanotechnology, various types of nanoparticles^1^, nanofibers^2^, nanotubes, nanorings^3^, nanoscrolls^4^ and nanosheets^5^ have emerged with promising applications for next generation electronics^6^,^7^, microchips^8^, composites^9^,^10^, biosensors, drug delivery^11^, and energy harvesting and conversion systems^12^. As a plate-like material consisting of a single layer of hexagonally arranged carbon atoms, graphene has attracted considerable attentions in the past decade because of its extraordinary physical and chemical characteristics^13^. Graphene is easily susceptible to out-of-plane deformation and tends to form wrinkles and folds during fabrication and application^14^,^15^,^16^. Patterned and hierarchy wrinkles and ripples have been observed and controlled in suspended graphene under both spontaneously and thermally generated strains^17^. Topological defects, such as disclinations (heptagons or pentagons) and dislocations (heptagon–pentagon dipoles), are also regarded as distributions inducing graphene wrinkling. An efficient continuum approach, which is capable of predicting the stress field and wrinkling patterns around disclinations and dislocations in graphene, has been developed^18^. These corrugations and wrinkles can be seen as a stability mechanism to relax the in-plane deformations of thin two dimensional materials^19^,^20^, and have been reported to affect the electronic and chemical properties of graphene by inducing effective magnetic fields^21^ and changing local potentials^22^,^23^,^24^. Among these wrinkled structures, annular graphene possesses special wrinkling patterns and shows a tremendous possibility to be used as flexible devices such as nano-force sensors, tunable magnetic or electronic devices, as well as patterned stretchable electronics in the future^25^,^26^,^27^. Various approaches such as molecular dynamics, molecular mechanics and continuum mechanics have been applied to study the wrinkle characteristic and torque capacity of circular graphene annulus under circular shearing. Zhang *et al*. studied the formation and characteristics of the wrinkling pattern in annular combing grillage model and molecular dynamics simulation^26^,^28^, and found unusual wrinkle patterns confined within a boundary layer instead of spreading throughout the entire material domain in rectangular graphene sheets. Zhao *et al*. investigated the effect of wrinkles on the surface area of graphene, and suggested that the high specific surface area of graphene can only be affected up to 2% regardless of loading conditions, geometry, and defects^29^. Li *et al*. reported the anomalous effect of wrinkling on the in-plane torque capacity of annulus by studying hydrogenated graphene annulus under circular shearing at the inner edge^30^. Wrinkling characteristics of graphene allotropes such as graphynes (*α-, β-, γ*-, and 6, 6, 12-graphyne) under in-plane circular shearing has also been studied^31^.

It is acknowledged that the mechanical response of circular and annular structures have strong dependency on boundary properties^32^,^33^,^34^. Understanding the boundary-dependent mechanical properties of annular graphene is essential for proper design of many MEMS (micro electro-mechanical systems) and NEMS (nano electro-mechanical system) devices. The wrinkle characteristics of circular graphene annulus including the wave number and amplitude have been reported to be sensitive to inner boundary radius^26^. However, limited knowledge is available about the effect of outer boundary on the mechanical properties and wrinkling characteristic of graphene annulus, which is in great need for its design and practical application. In this work, we first use MD simulations to study the effect of outer boundary on the mechanical properties and wrinkle characteristic of circular and elliptical graphene annulus under circular shearing at inner edge. The dependency of wrinkle characteristic and torque capacity on boundary are considered for a graphene annulus with varying inner radius, outer radius and aspect ratio. We are surprised to find that graphene annulus with elliptical outer boundary possess specific boundary-dependent wrinkle characteristic and torque capacity. Systematic discussions on the shear stress distribution and wrinkle characteristics including wave number, spiral angle and wave amplitude are carried out for the mechanism of outer boundary-dependent mechanical properties. Our results can be used for developing novel graphene -based devices.

## Results and Discussion

### Modeling of graphene annulus

**Figure 1:**
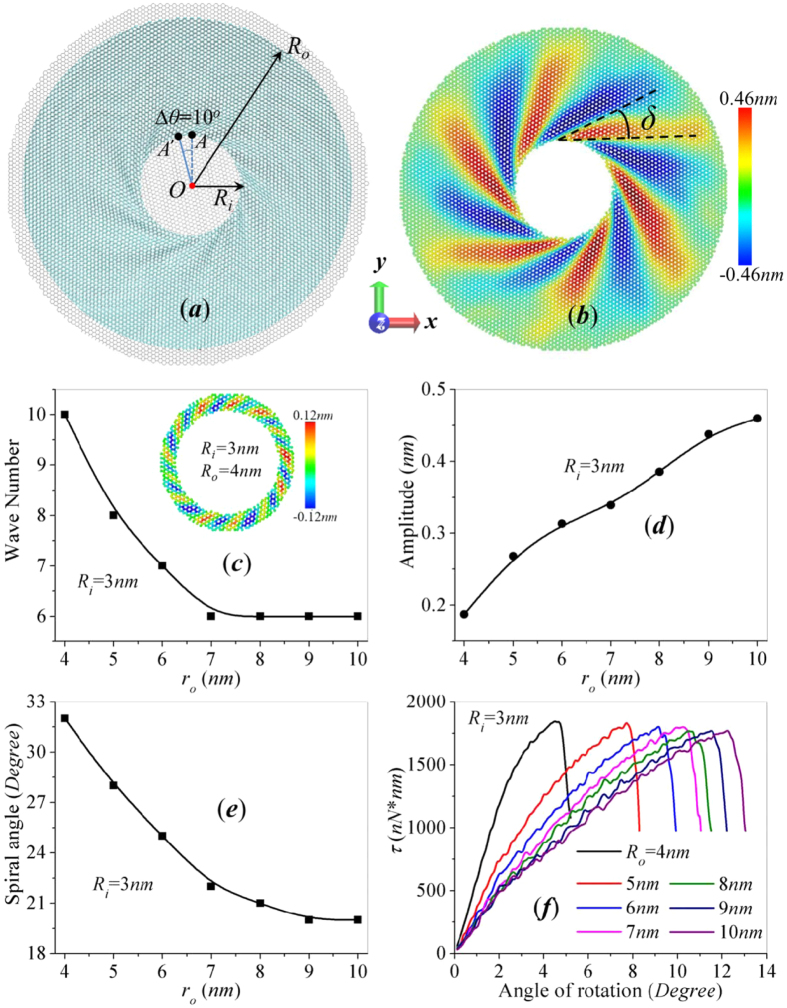
Circular graphene annulus subjected to in-plane rotation at inner edge with varying boundary radii *R*_i_, *R*_o_. (**a**) MD simulation snapshots of annulus with *R*_i_ = 3 *nm* and *R*_o_ = 10 *nm* under circular rotation at inner edge. The black point *A* at the inner edge serves as the reference point for rotation and the angle between the solid blue line and dash blue line illustrates the rotation angle *∆θ*. The gray domains represents the inner and outer boundaries which are not plotted in other figures for clarity. (**b**) Wrinkle amplitude contours of graphane annulus shown in (**a**) by coloring each atom according to the out-of-plane displacement. *δ* is the acute angle between the tangent of spiral wrinkle and the tangent of inner boundary through the tangent point. The contour in ***c*** represents the wrinkle profile of annulus with *R*_i_ = 3 *nm* and *R*_o_ = 4 *nm*. (**c–e**) The evolution of wrinkle characteristics with outer boundary radius *R*_*o*_. (**f**) Torque-torsional angle curves of annular graphene with fixed *R*_*i*_ = 3 *nm* and varying *R*_*o*_.

**Figure 2:**
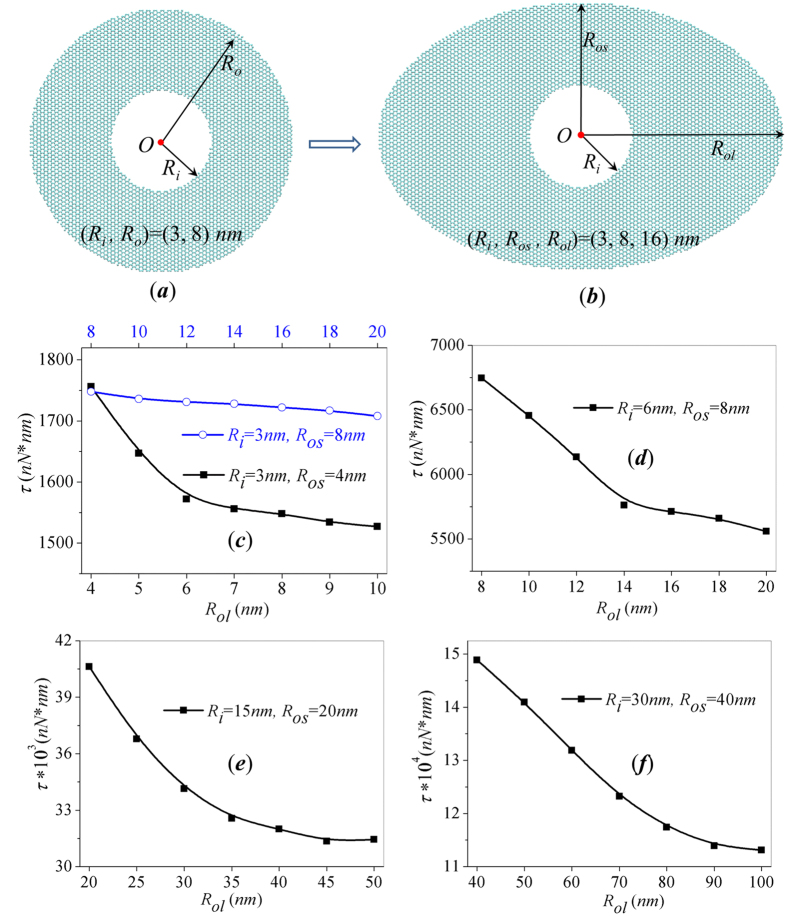
Effect of outer boundary aspect ratio on the torque capacity of elliptical graphene annulus. (**a,b**) Atomistic structure of elliptical annulus with (*R*_*i*_, *R*_*os*_, *R*_*ol*_) = (3, 8, 16) *nm* evolves from circular annulus with (*R*_*i*_, *R*_*o*_) = (3, 8) *nm*; (**c–f**) Evolution of torque capacity with the increase of major axis radius *R*_*ol*_ for annulus with fixed *R*_*i*_, *R*_*os*_.

The atomistic model of circular graphene annulus is shown in Fig. 1a. The atoms inside the radius *R*_*i*_ of the initial structure is defined as the inner boundary, and the atoms outside the radius *R*_*o*_ is the outer boundary. Boundary atoms are colored in gray while the free carbon atoms are colored in cyan. We also construct elliptical annulus with circular inner boundary and elliptical outer boundary featured with short axis *r*_*os*_ and long axis *r*_*ol*_, as shown in Fig. 2b. During relaxation process, boundary atoms are free to move in the plane of graphene annulus while the out-of-plane displacements are constrained by enforcing zero force and velocity along out-of-plane direction. The atoms between the inner and outer boundary are free to move during the whole simulation. After that, the atoms inside the inner boundary are rotated around the circle center as a rigid body at constant angular velocity 0.1 rad/*ps* until failure. The atoms of outer boundary are fixed during rotation by enforcing zero force and velocity. All sets of the simulation are performed at 300 K under NVT ensemble. The torque *M* imposed on the rigid rotating body, which equals to the torque in the domain *R*_*i*_ < *R* < *R*_*o*_, is recorded during the rotating process. The evolution of torque as a function of rotate angle is obtained to evaluate the torque capacity of annular graphene. Since there is no experimental result about the strength of graphene under in-plane shearing in literature, we compare our results with reported simulation results in literatures for verification. We calculated the torque-angle of rotation (*τ*-∆*θ*) curve for circular graphene annulus with *R*_*i*_ = 1.5 *nm*, *R*_*i*_ = 4.5 *nm* which has been studied by Qin *et al*.^35^. Our recorded *τ*-∆*θ* curve shows a peak value of 541 nN*nm as well as three turning points at ∆*θ*_1_ = 3°, ∆*θ*_2_ = 11°, ∆*θ*_3_ = 17° (as plotted in Fig. S4, these three turning points correspond to the moment of wrinkle initiation, failure initiation, complete failure), which are consistent with the reported peak value 592 nN*nm and ∆*θ*_1_ = 4°, ∆*θ*_2_ = 9°, ∆*θ*_3_ = 15° by Qin *et al*. Furthermore, we applied out model for the wrinkle characteristics of circular graphene annulus with *R*_*i*_ = 3 *nm*, *R*_*o*_ = 9 *nm* at 1 *K* which has been studied by Tian *et al*.^31^. The wrinkle profile at critical rotational angle is calculated to contain 8 wrinkle waves with wrinkle amplitude of 0.27 nm (as shown in Fig. S5), which agrees well with the results of Tian *et al*. (8 wrinkle waves and wrinkle amplitude 0.26 *nm*). The agreement confirms that our MD simulations are appropriate and reliable. Our recent work on the torque capacity of surface functionalized graphene annulus also adopts the same simulation procedures described above^30^.

### Boundary radius-dependent wrinkling and torque capability of circular annulus

Under in-plane circular shearing, the annular graphene remains flat when the rotational angle is small. After inner boundary rotate through some angle ∆*θ*, the annulus show out-of-plane buckling suddenly and a series of spiral wrinkles concentrated around the inner rim of the annulus are noticed. These wrinkles decay along the radial direction, indicating a transition from bending to stretching in the annular domain. The wave amplitude and length of wrinkles increases gradually with the increase of rotational angle. When the applied rotation reaches critical rotational angle *∆θ*_*c*_, the material starts to fail due to the break of covalent bonds on the inner boundary. Eventually, the wrinkle pattern vanishes (as illustrated in Videos S1 and S2).

Figure 1a shows the snapshots from the dynamic rotation of circular annulus at critical torsional angle with *R*_*o*_ = 10 *nm* and *R*_*i*_ = 3 *nm*. Figure 1b is the corresponding wrinkle contour of by coloring each atom according to the displacement in the out-of-plane direction, and the difference in color intensity reveals the difference in wrinkle amplitude. Such wrinkling pattern can be characterized by wave number, wrinkle amplitude and wrinkle spiral angle which dependents on the boundary radius^26^,^29^,^30^. The spiral angle *δ* is the acute angle between the tangent of spiral wrinkle and the tangent of inner boundary through the tangent point as illustrated in Fig. 1b. What’s more, the wrinkle profile plotted in Fig. 1b shows *a* spiral angle of *δ* = 19° which also agrees well with the value 20° reported by Wang *et al*.^32^ and the value 18° reported by Zhang *et al*.^26^.

The dependency of mechanical properties on outer boundary radius is considered by constructing circular annulus with varying *R*_*o*_ and *R*_*i*_. Figure 1c–e shows the change of wave number, wrinkle amplitude and spiral angle for circular annulus with fixed *R*_*i*_ = 3 *nm* and *R*_*o*_ ranging from 4–10 *nm*. The wrinkle amplitude increase almost linearly with the outer radius, while the wave number and spiral angle both decrease sharply with *R*_*o*_ and reach constant when *R*_*o*_ is large enough. All the wrinkle characteristics of circular annulus show strong sensitivity to the increase of outer radius *R*_*o*_. Figure 1b and c shows the wrinkle contours of annuli with same inner radius *R*_*i*_ = 3 nm but different outer radius *R*_*o*_. For thin circular annulus with *R*_*o*_ = 4 nm shown in Fig. 1c, the wrinkling profiles under circular shear spreads throughout the annulus. The wrinkle pattern is similar to that of graphene nanoribbons under pure shear as reported by Wang *et al*.^36^. With the increase of outer radius, the wrinkle profile under circular shearing gradually transform to spiral wrinkling pattern surrounding the inner edge (Fig. 1b). The out-of-plane corrugation of the spiral wrinkles decays along the radial direction of annulus. Thus, the effect of outer boundary on the wave characteristics decreases with the increase of outer radius because of such decaying spiral wrinkling pattern. As plotted in Fig. 1c and e, the wave number and the spiral angle decrease with the increase of *R*_*o*_ and reach a stable value when *R*_*o*_ is large enough. Figure 1d shows the evolution of torque capacity as a function of the torsional angle. By studying the torque-torsional angle (*τ*-*∆θ*) curves, the slope of all curves suddenly drops when *∆θ* approximate to 3°, indicating the initiation of wrinkles. With the increase of outer radius, the critical torsional angle *∆θ*_*c*_ increases without sacrificing the torque capacity of circular annulus, i.e. the peak value of these curves. The torsional stiffness also reaches stable when the radii ratio *R*_*o*_/*R*_*i*_ is large enough. We also investigate the effect of inner radius by constructing circular annulus with fixed *R*_*o*_ = 20 *nm* and *R*_*i*_ = 1, 2, 3, 4, 5 *nm*, and the corresponding *τ*-*∆θ* curves are calculated as shown in Fig. S1a. For annulus with large radii ratio *R*_*o*_/*R*_*i*_ and fixed *R*_*o*_, the critical torsional angle decreases with the increase of inner radius while the torque capacity increases with *R*_*i*_. All the peak values of these curves gives the same material strength by dividing 2*π*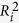, the increase of torque capacity with *R*_*i*_ is caused by the increase of effective contact area between annulus and the rotating inner boundary. Figure S1b shows the evolution of wrinkle amplitude and wave number on the inner radius of circular annulus. With the increase of *R*_*i*_, wrinkle amplitude increase exponentially to a stable magnitude while wave number increases almost linearly as reported in literature^26^.

The advancement of fabrication technology permits graphene nanosheets to be tailored into designed shapes for nano-devices. To better prepare annular graphene for practical application, we constructed annulus with varying outer boundary for the shape effect of outer boundary on torque capacity of graphene annulus as shown in Fig. S6a and b. By calculating the torque and wave number of square and equilateral triangular graphene annulus with fixed *R*_*i*_ and varying *R*_*o*_. The variation of mechanical properties with the increase of outer radius *R*_*o*_ shows the same trend with circular annulus (More details can be referred in SI). Thus the shape effect of outer boundary on the mechanical properties of graphene annulus under in-plane circular shearing can be revealed effectively by studying the annulus with circular outer boundary.

Since our simulations focus on the effect of outer radius, we also consider another loading protocol by applying in-plane rotation at outer edge while keeping the inner edge fixed. The described simulations in Fig. 1 are repeated under such loading protocol and the torque capacity and wrinkle features for circular annulus with different outer radius are obtained as shown in Fig. S2. By comparing the mechanical properties of circular and elliptical annulus under these two different loading protocols, the difference between these two loading protocols are shown to be negligible while the results of annulus under rotating at inner edge are more smooth and stable. Thus, we adopted the loading protocol with fixed outer boundary and rotating inner edge in the following part. More detailed discussions can be referred in SI.

### Torque capacity of elliptical graphene annulus

Elliptical structures widely exist in engineering structures, circuits and electrical devices. To better prepare annular graphene for application, we also constructed annulus with elliptical outer boundary for the shape effect of outer boundary on torque capacity of graphene annulus. The outer boundary of elliptical annulus can be described with feature parameters, minor-axis radius *R*_*os*_ and major-axis radius *R*_*ol*_. Figure 2a,b shows the comparison of circular and elliptical annulus with same inner boundary radius *R*_*i*_ = 3 *nm*, and *R*_*o*_ = *R*_*os*_ = 8 *nm*. By repeating the simulation described in Fig. 1f, we can get the *τ*-*∆θ* curves and the torque capacity for elliptical annulus under circular rotation at inner edge. Figure 2c shows the calculated torque capacity of elliptical annuli with different aspect ratios. The black line in Fig. 2c represents annuli with *R*_*i*_ = 3 *nm*, *R*_*os*_ = 4 *nm* and varying *R*_*ol*_ in the range of 4–10 *nm*. With the increase of the aspect ratio between *R*_*ol*_/*R*_*os*_, the outer boundary gradually transforms from circle to ellipse. The torque capacity first decrease sharply with the increase of aspect ratio *R*_*ol*_/*R*_*os*_ and reaches stable when *R*_*ol*_/*R*_*os*_ > 2.5. The decrease of torque capacity caused by the change of boundary shape can be up to 20%.

Existing literatures on the torsional properties of annular graphene is based on MD models less than 10 nm, while the application range of nanomaterials in such small size is limited. Therefore, it is necessary to check whether the shape effects still exist in graphene sheets with larger scale. In order to verify the generality of the boundary shape effect, we enlarge the size of elliptical annulus by 2, 5 and 10 times separately and repeat the described simulations. As shown in Fig. 2d–f, the torque capacity of the enlarged annuli all shows similar decay trend with the outer boundary aspect ratio. The largest decay of torque capacity exceeds 20%. The black lines in Fig. 2c–f demonstrate that the shape effects of outer boundary are applicable for annulus in micro scale.

The blue line in Fig. 2c represents the torque capacity of another group of annulus with fixed *R*_*i*_ = 3 *nm*, *R*_*os*_ = 8 *nm* and varying *R*_*ol*_ in the range of 8–20 *nm*. It is interesting to notice that the largest decay of torque capacity caused by the change of aspect ratio is less than 3%. Thus, the torque capacity of annulus with large radii ratio *R*_*os*_/*R*_*i*_ is almost insensitive to the change of outer boundary shape. The difference between the blue line and black line implies a coupled effect of boundary shape and radii ratio *R*_*os*_/*R*_*i*_ (annulus thickness) on torque capacity. For annuli with small radii ratio *R*_*os*_/*R*_*i*_ (thin annulus), the torque capacity decrease obviously with the increase of major axis *R*_*ol*_, while annuli with large radii ratio *R*_*os*_/*R*_*i*_(thick annulus) has shape-independent torque capacity.

We further compared the torque capacity of graphene annulus with elliptical and rectangular outer boundaries. The outer boundary of elliptical and rectangular annulus can be described with feature parameters, minor-axis radius *R*_*os*_ and major-axis radius *R*_*ol*_ as illustrated in Fig. S7. By calculating the evolution of torque capacity with the increase of major axis radius *R*_*ol*_ for annulus with fixed *R*_*i*_, *R*_*os*_, we noticed that the results of annulus with rectangular outer boundary is constant with the results elliptical annulus with same featured radius. Thus, we choose elliptical annulus, whose wrinkle features and stress distribution can be described more conveniently, as a typical geometry for the effects of boundary aspect ratio and radii ratio *R*_*os*_/*R*_*i*_ on torque capacity.

**Figure 3:**
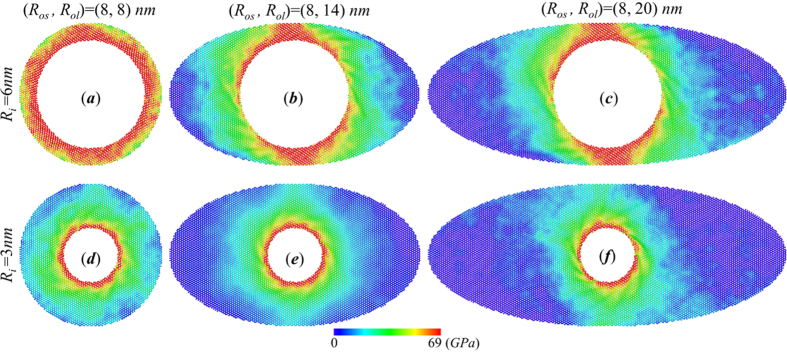
Coupled effect of outer boundary shape and radii ratio *R*_*os*_/*R*_*i*_ on shear stress distribution of elliptic annular graphene. (**a–c**) Shear stress contours of annuli with fixed *R*_*i*_ = 6 *nm*, *R*_*os*_ = 8 *nm* and varying *R*_*ol*_ = 8, 14, 16 *nm* under critical torsional angle. (**d–f**) Shear stress contours of annuli with fixed *R*_*i*_ = 3 *nm*, *R*_*os*_ = 8 *nm* and varying *R*_*ol*_ = 8, 14, 16 *nm* under critical torsional angle.

In order to explain the mechanism for the coupled effect of boundary aspect ratio and radii ratio *R*_*os*_/*R*_*i*_ on torque capacity, we further calculate the in-plane shear stress distribution of elliptical annulus under critical torsion angle. Figure 3 shows the comparison of shear stress contour for two groups of annuli with *R*_*i*_ = 6 nm and *R*_*i*_ = 3 nm. For circular annuli (Fig. 3a,d), the stress distribution is relatively uniform around the inner edge, regardless of the outer radius *R*_*o*_. Since the failure of annuli is caused by the stress concentration around the inner edge, the torque capacity of annulus with same inner radius is close, as demonstrated by Fig. 2c (the first points of black and blue lines). With the increase of major axis *R*_*ol*_, the stress distribution of thick elliptical annuli with *R*_*os*_ = 8 *nm*, *R*_*i*_ = 3 *nm* (Fig. 3a–c) is almost as uniform as circular annulus, as a result, the torque capacity does not change much with *R*_*ol*_. For thin annuli with fixed *R*_*os*_ = 8 *nm*, *R*_*i*_ = 6 *nm* (Fig. 3d–f), the stress concentration near the short axis becomes more and more severe with *R*_*ol*_. As a result, the torque capacity of the elliptical structure decays with the increase of the aspect ratio. However, the increase of aspect ratio will not lead to consistent decrease of *τ*. By comparing Fig. 3b and c, we find that the stress concentration degree converge to a stable state after *R*_*ol*_ is large enough, leading to the decrease of decay rate.

### Wrinkling characteristics of elliptical graphene annulus

**Figure 4:**
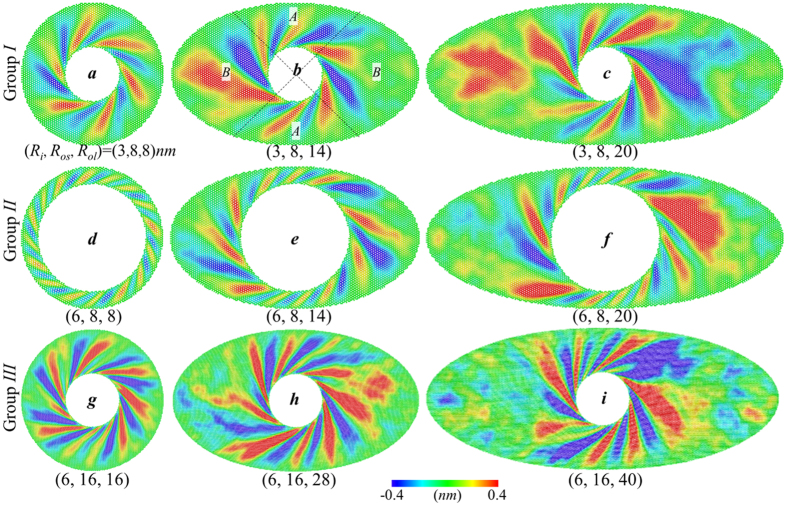
Coupled effect of outer boundary shape and radii ratio *R*_*os*_/*R*_*i*_ on wrinkling characteristics of elliptic graphene annulus. The wrinkle contours are colored based on the out-of-plane deformation under critical rotation angle *∆θ*_*c*_ for annuli with varying (*R*_*i*_, *R*_*os*_, *R*_*ol*_). (**a–c**) Annuli in group *I* have fixed *R*_*i*_ = 3 *nm*, *R*_*os*_ = 8 *nm* and varying *R*_*ol*_ = 8, 14, 20 *nm*, (**d–f**) group *II* have fixed *R*_*i*_ = 6 *nm*, *R*_*os*_ = 8 *nm* and varying *R*_*ol*_ = 8, 14, 20 *nm*, (**g–i**) group *III* have fixed *R*_*i*_ = 6 *nm* and same radii ratio *R*_*os*_/*R*_*i*_ as group *I*. The annuli are divided into two symmetrical domains *A* and *B* which represent the area close to miner axis and major axis respectively.

Based on the dynamic rotating process (Video S2), boundary shape of annuls also show noticeable influence on the wrinkling characteristics of annular graphene. Figure 4 shows the wrinkle contour of three groups of graphene annuli under critical rotation angle with varying (*R*_*i*_, *R*_*os*_, *R*_*ol*_). Group *I* and Group *III* are thick annuli which have same radii ratio *R*_*os*_/*R*_*i*_ while Group *II* belongs to thin annulus with small radii ratio *R*_*os*_/*R*_*i*_. The result also shows a coupled effect of boundary shape and radii ratio *R*_*os*_/*R*_*i*_ on wrinkling characteristics. Wrinkle profiles in group *II* change obviously with the increase major axis radius, especially for these wrinkles close to the major axis.

To better understand the effects of boundary aspect ratio on the wrinkling characteristics of elliptical annulus, we divide the annular graphene into two pair of symmetrical sectors *A* and *B* as illustrate in Fig. 4b. Sector *A* represents the domain close to miner axis while sector *B* represents the region of major axis. The wave number and spiral angle of the wrinkles in each sector of annuli are measured for comparison. Table 1 shows the characteristics of wrinkles in sector *A* and *B* for three groups of annulus. The listed spiral angle is the average value of all wrinkles in sectors *A* or *B*. Group *I* and *III* are thick annuli with same radii ratio *R*_*os*_/*R*_*i*_ = 8/3 while Group *II* are thin annuli with *R*_*os*_/*R*_*i*_ = 4/3. Due to the axial symmetry of wrinkle pattern in circular annuli, wrinkles in sector *A* and sector *B* have equal wave number and spiral angle when *R*_*ol*_ = *R*_*os*_. With the increase of major axis radius, the wave number and spiral angle of wrinkles in sector *A* and *B* is basically unchanged for the annuli in group *I* as well as group *III* (which is twice the size of group *I*). However, there is an obvious divergence between wrinkle characteristics in sector *A* and sector *B* for group *II* when *R*_*ol*_ varies from 8 *nm* to 20 *nm*. The wave number and spiral angle of wrinkles in domain *B* show significantly change with the increase of boundary aspect ratio while the wrinkle characteristics in domain *A* are basically unchanged. Both the wave number and spiral angle of wrinkles in sector B of group *II* decrease with *R*_*ol*_ and reach steady state after aspect ratio *R*_*ol*_/*R*_*os*_ ≥ 2. Since all these wrinkle characteristics are listed in Table 1 are measured at critical rotation angle, we also record the critical angle ∆*θ*_*c*_ for all these annuli. The recorded ∆*θ*_*c*_ reaches 12° for both the annuli in group *I* as well as group *III* while it is just about 6° for the annuli in group *II*. The critical rotation angle of elliptical annulus is found to be independent on the radii ratio *R*_*os*_/*R*_*i*_ but dependent on radii ratio *R*_*ol*_/*R*_*os*_. The recorded ∆*θ*_*c*_ also demonstrate that the torsional stiffness and torque capacity show different dependency on boundary aspect ratio of elliptical annulus. Thus, we conclude that the torque capacity of annulus is not directly related to the wrinkle stiffness. What’s more, it is interesting to notice that the dependency of wrinkle characteristics on boundary shape is similar to that of torque capacity, which reveals the wrinkling characteristics of annular structures should account for the stress distribution of the annular graphene, detailed specific mechanism of out-of-plane deformation on in-plane torque capacity has been reported in ref. 30.

Furthermore, compared with the wrinkle characteristic of circular graphene annulus, we have more interesting observations in the wrinkle profiles in domains *A* and *B*. For group *II,* the wave number and spiral angle of wrinkles in domain *A* are basically unchanged and the values are close to those in circular graphene annulus with same *R*_*o*_ and *R*_*i*_ (*R*_*i*_ = *R*_*os*_). While the wrinkles in domain *B* show significantly change with the increase of *R*_*ls*_, which is similar to the described phenomenon in circular graphene that the wave number and spiral angle first decrease sharply with *R*_*o*_ and reaches constant with the increasing outer radius. What’s more, the change of wrinkle characteristics with outer boundary radius is trivial when *R*_*o*_ is large enough in circular graphene. This feature could explain the phenomenon that wave number and spiral angle of wrinkles in sector *A* and *B* are almost remain the same for the annuli in group *I* as well as group *III*.

Therefore, we believe that domain *A*, *B* in elliptical annular graphene have similarities with circular graphene annulus with same *R*_*o*_ and *R*_*i*_ (*R*_*i*_* = R*_*os*_ or *R*_*i*_* = R*_*ls*_) on the mechanical properties and wrinkle characteristic. Based on this hypothesis, we can give an explanation to the stress distribution of the elliptical annular graphene. For the thin annuli, domain *A* possesses low critical rotation angle ∆*θ*_*c*_ while domain *B* owns higher ∆*θ*_*c*_with increasing *R*_*ls*_ (the critical torsion angle *∆θ*_*c*_ increases outer radius). During the dynamic rotating process, domain *A* would firstly reach critical rotation angle and failure, leading to high stress concentration in domain *A*. What’s more, with the increase of boundary aspect ratio, the difference of ∆*θ*_*c*_ between domain *A* and *B* would reach a stable level (the critical torsion angle *∆θ*_*c*_ reach stable when the radii ratio *R*_*o*_/*R*_*i*_ is large enough), that is why the torque capacity of elliptical graphene annular is basically unchanged when *R*_*o*_/*R*_*i*_ is large enough. However, for thick annuli with large radii ratio *R*_*os*_*/R*_*i*_, the domain *A* and *B* possess similar ∆*θ*_*c*_ which resulting in similar stress concentration in domain *A* and *B*. The recorded ∆*θ*_*c*_ also support such hypothesis. The ∆*θ*_*c*_ in group *I* and group *III* reaches 12° which is same with the value of circular graphene annular with large outer radius. The ∆*θ*_*c*_ in group *II* is 6° which equals to the value of thin circular graphene annular (*R*_i_ = 3 *nm*, *R*_o_ = 4 *nm*).

**Figure 5:**
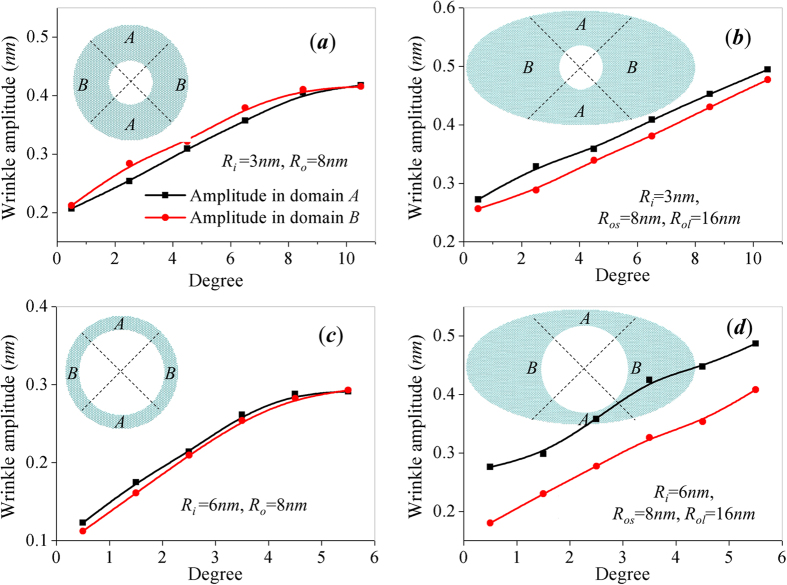
Evolution of wrinkle amplitude during the dynamic rotating process of circular and elliptic graphene annuli with different boundary radius and radii ratio. Each annuli are divided into two symmetrical sectors *A* and *B*, and the black solid line represents wrinkles amplitude in sectors A while the red dash lines represents wrinkles amplitude in sectors B. (**a**) Circular annulus with radius *R*_*i*_ = 3 *nm, R*_*o*_ = 8 *nm*, (**b**) elliptical annulus with radius *R*_*i*_ = 3 *nm, R*_*os*_ = 8 *nm*, *R*_*os*_ = 16 *nm*, (**c**) Circular annulus with radius *R*_*i*_ = 6 *nm, R*_*o*_ = 8 *nm*, (**d**) elliptical annulus with radius *R*_*i*_ = 6 *nm, R*_*os*_ = 8 *nm*, *R*_*os*_ = 16 *nm*.

We also record the evolution of wrinkle amplitude during the rotation process to further discuss the effect of boundary shape on wrinkling characteristics. Figure 5 shows the comparison of wrinkle amplitude in circular and elliptical annulus with same inner boundary, the minor-axis radius of elliptical annulus equals to the outer radius of circular annulus. We still split the annuli into *A*, *B* domains for discussion, and the plotted wrinkle amplitude in domain *A*, *B* is taken as the maximum out-of-plane deformation of the wrinkles in each domain. As we expected, the wrinkle amplitude of domain *A* equals to that of domain *B* in circular annulus as demonstrated. For thick elliptical annuli with large radii ratio *R*_*os*_/*R*_*i*_ = 8/3 (Fig. 5b), the wave amplitude in the area *A* equal to that of wrinkles in the area *B* during the whole rotating process. For elliptical annuli with relative smaller *R*_*os*_/*R*_*i*_ = 4/3 (Fig. 5d), the wrinkles in area *A* and *B* have wave amplitude at the beginning of the rotation process. However, wave amplitude becomes uniform with the increase of rotating angle. The amplitude of wrinkles close to major-axis grows faster than that of wrinkles close to minor amplitude. Figure 5 shows there is also a coupled effect of boundary aspect ratio and radii ratio *R*_*os*_/*R*_*i*_ on wrinkle amplitude which is one of the most important wrinkling characteristics. Larger wrinkle amplitude has been reported to contribute to the torque capacity^30^. Therefore, uniform wave amplitude should account for the stress concentration, which is also an important factor resulting in the decay of torque capacity of elliptical annular graphene.

In conclusion, the effect of outer boundary radius and aspect ratio on the wrinkling-related mechanical properties of annular graphene under rotation at inner boundary has been systematically studied. For circular graphene annulus, both the torque capacity and critical rotation angle can be effectively tuned by the boundary radius. The critical rotating angle of circular annulus with fixed inner radius can be increased in several times by increasing the radius of out boundary without sacrificing torque capacity. The wave number of wrinkles in circular annulus also increases with outer radius accompanied by a threshold value beyond which the wave number becomes constant. Elliptical annuli with varying aspect ratios are studied for the dependency of wrinkling and torsion characteristics on the shape of outer boundary. A coupled effect of the ratio of major axis to minor axis and the ratio of minor axis to inner radius are revealed for the wrinkling related mechanical properties of elliptical annulus. The torque capacity of elliptical annulus with a large ratio of minor axis to inner radius is found to decrease with the increase of aspect ratio, and the decay could be up to 20%. The dependency of wrinkle characteristics on boundary shape is similarity to that of torque capacity, which demonstrates that the wrinkling characteristics of graphene annulus are closely related to the torque capacity.

By studying the shear stress distribution and wrinkle characteristics including wave number, spiral angle and wave amplitude of elliptical annulus, we find the decay of torque capacity with the increase of major axis is the result of shear stress concentration around the minor axis, such nonuniform stress distribution is anomalously caused by the change of wrinkle profiles near the major axis. Our results demonstrate that the topological and mechanical characteristics of graphene annulus can be tailored with boundary properties such as size and aspect ratio, the specific mechanism of out-of-plane deformation on in-plane strength opens up a straightforward means to develop novel graphene -based devices.

## Methods

### Molecular dynamics simulation

We adopt the Adaptive Intermolecular Reactive Empirical Bond Order (AIREBO) force field^37^ in LAMMPS package^38^ to model the carbon-carbon interactions of graphene annulus. Circular and elliptical graphene annuli are described in main text. Prior to loading, the boundary atoms are relaxed for 2000 MD steps with time step *τ = *0.1 *fs*, followed by another relaxation of 5000 MD steps with *t*_*step*_* = *1*fs*. During relaxation process, boundary atoms are free to move in the plane of graphene annulus while the out-of-plane displacements are constrained by enforcing zero force and velocity along out-of-plane direction. The atoms between the inner and outer boundary are free to move during the whole simulation. After that, the atoms inside the inner boundary are rotated around the circle center as a rigid body at constant angular velocity 0.1 rad/*ps* until failure. The atoms of outer boundary are fixed during rotation by enforcing zero force and velocity.

The torque *M* imposed on the rigid rotating body equals to the torque in the domain *R*_*i*_ < *R* < *R*_*o*_. Thus the torque on the graphene annulus is recorded by calculating the torque on the rigid domain *R*_*i*_ > *R* during the rotating process. During simulation, we tread the rigid rotating body as a group and calculate the torque in the rigid body using the command “variable name equal torque (group, dim)” where “torque (group, dim)” represents a group function calculating the torque applying on a group of atoms. All sets of the simulation were performed at room temperature under NVT ensemble.

## Additional Information

**How to cite this article**: Li, Y. *et al*. Boundary-dependent mechanical properties of graphene annular under in-plane circular shearing via atomistic simulations. *Sci. Rep.* **7**, 41767; doi: 10.1038/srep41767 (2017).

**Publisher's note:** Springer Nature remains neutral with regard to jurisdictional claims in published maps and institutional affiliations.

## Supplementary Material

Supplementary Information

Supplementary Video S1

Supplementary Video S2

Supplementary Video S3

## Figures and Tables

**Table 1:** The change of wrinkling characteristics and critical torsion angle ∆*θ*_*c*_ with major axis radius *R*_*ol*_for three groups of elliptical annulus with varying (*R*_*i*_, *R*_*os*_, *R*_*ol*_).

*R*_*ol*_ (*nm*)			8	10	12	14	16	18	20
Domain									
*R*_*i*_ = 3*, R*_*os*_ = 8	*N*	*A*	3	3	3	3	3	3	3
		*B*	3	3	3	3	3	3	3
	*δ*	*A*	21°	21°	20°	21°	21°	20°	20°
		*B*	21°	20°	20°	19°	20°	20°	19°
	∆*θ*_*c*_	*A*&*B*	10.8°	11.6°	11.7°	12.4°	12°	11.7°	12.6°
*R*_*i*_ = 6*, R*_*os*_ = 8	*N*	*A*	8	8	7	7	7	7	7
		*B*	8	6	5	4	4	4	4
	*δ*	*A*	31°	32°	30°	31°	31°	30°	30°
		*B*	31°	28°	25°	23°	22°	22°	21°
	∆*θ*_*c*_	*A*&*B*	5.4°	5.8°	5.9°	5.9°	6.1°	6°	6.3°
**Domain**			**16**	**20**	**24**	**28**	**32**	**36**	**40**
**R_ol_ (nm)**									
*R*_*i*_ = 6*, R*_*os*_ = 16	*N*	*A*	5	5	5	5	4	4	4
		*B*	5	5	5	4	4	4	4
	*δ*	*A*	21°	21°	21°	20°	19°	19°	20°
		*B*	21°	22°	22°	21°	21°	21°	22°
	∆*θ*_*c*_	*A*&*B*	11.9°	12.4°	12.3°	12.3°	12°	12.4°	12.8°

The wrinkling characteristics including wrinkle number *N* and spiral angle *δ* are measured for wrinkles in domain A and domain B for discussion. The listed spiral angle is the mean value of all wrinkles in each domain.
