# Supplementary material for: Boundary-dependent mechanical properties of graphene annular under in-plane circular shearing via atomistic simulations
Source: Sci Rep. 2017 Feb 13;7:41767. doi: 10.1038/srep41767 (PMC5304194; doi:10.1038/srep41767)
Supplement: Supplementary Information [file srep41767-s1.doc]

**SUPPLEMENTARY INFORMATION**

Boundary-dependent mechanical properties of graphene annular under in-plane circular shearing via atomistic simulations

Yinfeng Li^1,*^, Qianling Lin^1^, Daxiang Cui^2,*^

^1^ Department of Engineering Mechanics, School of Naval Architecture, Ocean and Civil Engineering (State Key Laboratory of Ocean Engineering, Collaborative Innovation Center for Advanced Ship and Deep-Sea Exploration), Shanghai Jiao Tong University, Shanghai 200240, China

^2^ Institute of Nano Biomedicine and Engineering, Key Laboratory for Thin Film and Microfabrication Technology of the Ministry of Education, Department of Instrument Science and Engineering, School of Electronic Information and Electrical Engineering, Shanghai Jiao Tong University, Shanghai 200240, China

**The Electronic Supplementary Information (ESI) includes three videos:**

**Video S1**: The dynamic simulation process (including preliminary relaxation by energy minimization, further relaxation, rotation process) of annular graphene annulus under circular rotating at inner edge with *R_i_*=3*nm*, *R_o_*=8*nm*.

**Video S2**: The dynamic simulation process of elliptical graphene annulus under circular rotating at inner edge with inner radius *R_i_*=3*nm*, *R_os_*=8*nm* and *R_ol_*=12*nm*.

**Video S3**: The dynamic simulation process of elliptical graphene annulus under circular rotating at inner edge with inner radius *R_i_*=3*nm*, *R_os_*=8*nm* and *R_ol_*=20*nm*.


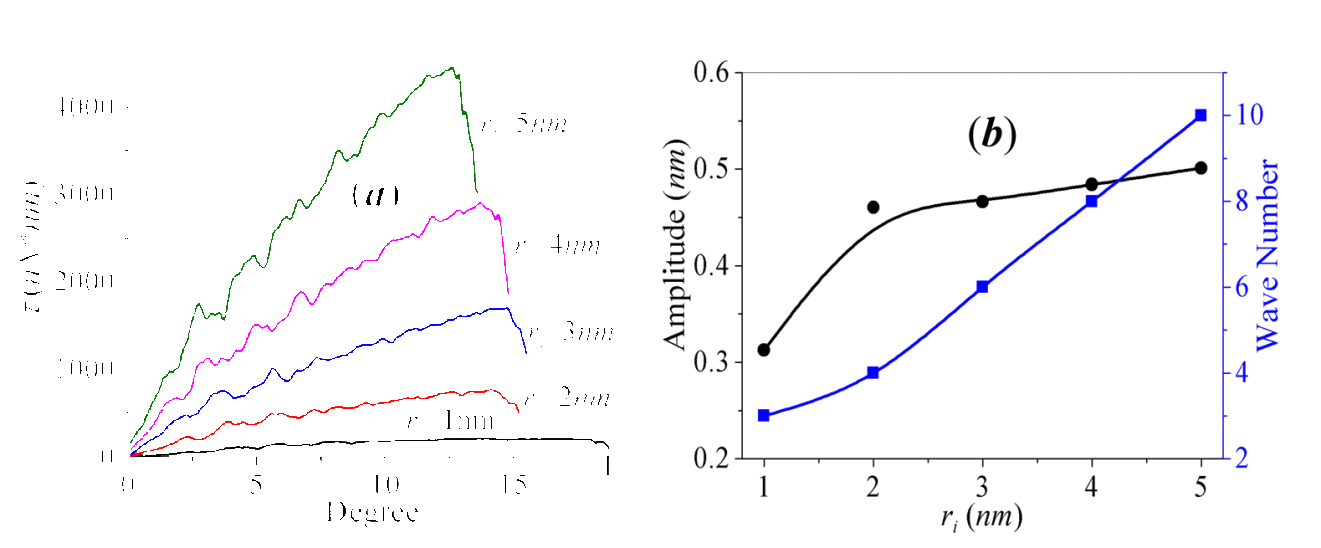


**Figure S1 | Circular graphene annulus subjected to in-plane rotation at inner edge with varying boundary radii *R_i_*, *R_o_*.** (***a***) The torque-torsion angle curves of annular graphene with fixed *R_o_*=10*nm* and varying *R_i_*. (***b***) The evolution of wrinkle characteristics with outer radius *R_o_*.

Figure S2 and S3 show the comparisons of the mechanical properties under shearing at inner edge and shearing at outer edge for circular and elliptical annulus, respectively. It can be noticed the difference in wrinkling characterizes and torque capacity between these two loading protocols are negligible as expected. The equivalent of these two loading protocol are reasonable because the suggested loading protocol take inner boundary as reference object while our adopted loading protocol regards outer boundary as reference object. The rotation at outer edge is equivalent to same amount of rotation at inner edge. However, the results also show that in-plane rotation at outer edge causes more severe fluctuation in the graphene membrane. Figure S3*a* shows the torque-torsional angle curves of circular graphene annulus with fixed inner edge *R_i_*=3*nm* and *R_o_*=4*nm* under in-plane rotation at outer edge. Compared to the torque-torsional angle curves plotted in Fig. 1 of the main text, the results of annulus under circular shearing at inner edge is more stable and smooth.


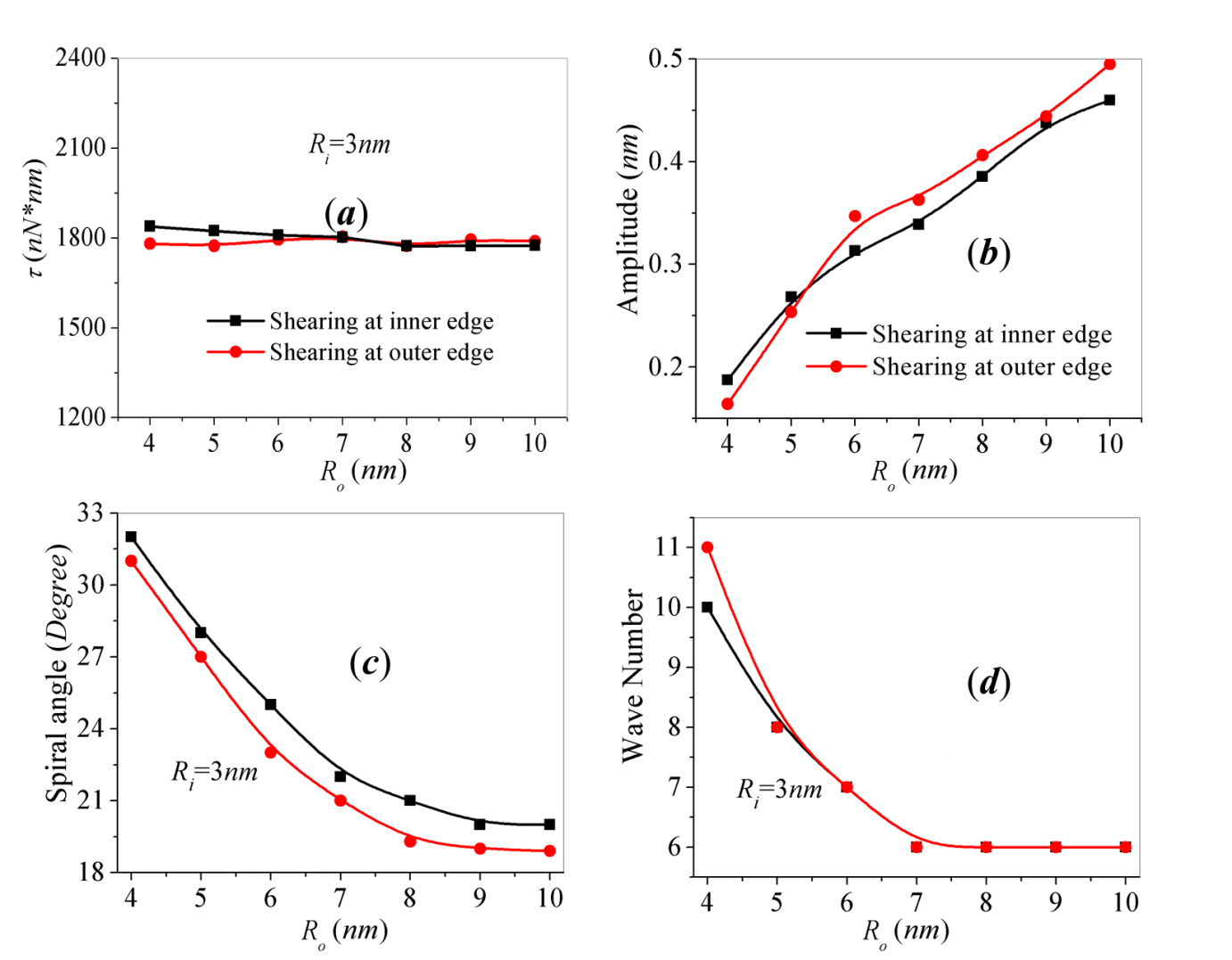


**Figure S2 | Mechanical properties of circular graphene annulus with boundary radii *R*_i_=3 and varying *R*_o_ under two different loading protocols.** The red lines shows the results of annulus with fixed inner edge under in-plane rotation at outer boundary while the black lines represents the results of annulus with fixed outer edge under in-plane rotation at inner edge. (***a***) Torque capacity of annular graphene with fixed *R_i_*=3*nm* and varying *R_o_*. (***b-d***) The evolution of wrinkle characteristics with outer boundary radius *R_o_*.


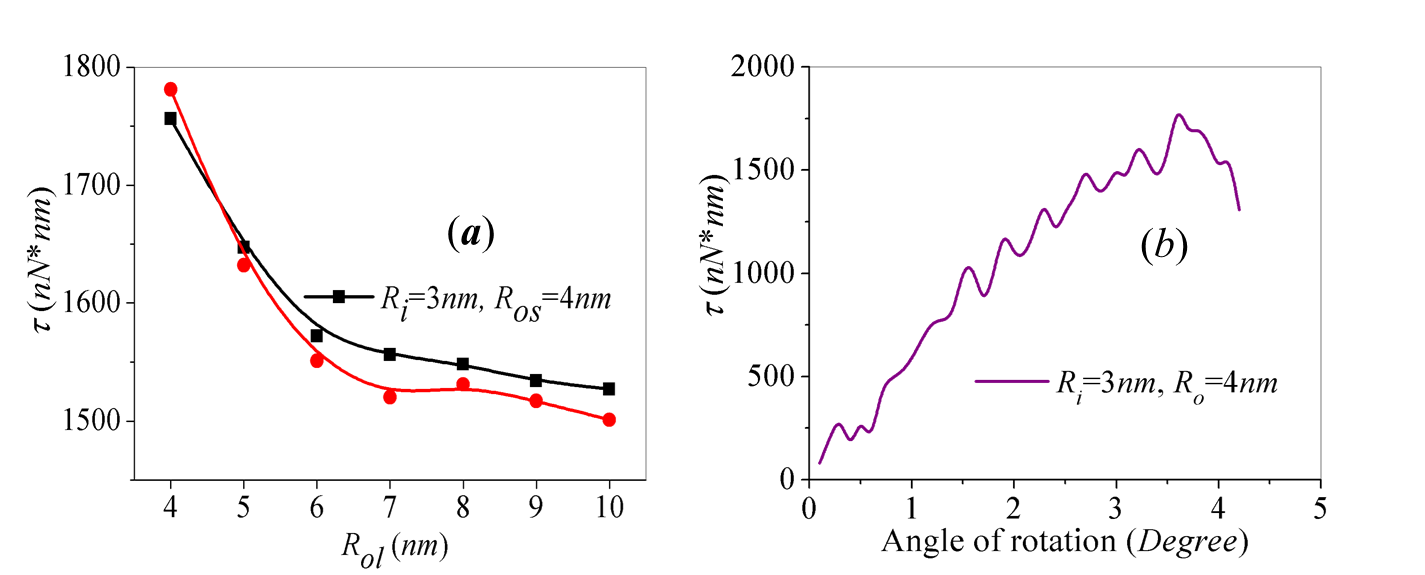


**Figure S3 | Torque capacity of circular graphene annulus with boundary radii *R*_i_=3, *R_ol_*=4*nm* and varying *R_ol_* under two different loading protocols.** (*a*) The red lines shows the results of annulus with fixed inner edge under in-plane rotation at outer boundary while the black lines represents the results of annulus with fixed outer edge under in-plane rotation at inner edge. (*b*) Torque-torsional angle curves of circular graphene annulus with fixed inner edge under in-plane rotation at outer edge.

The accuracy of our model is verified by comparing our results with reported simulation results in literatures. We first constructed the graphene annulus with *R_i_*=1.5*nm*, *R_o_*=4.5*nm* which is same as the model considered by Qin *et al* [1]. Our calculated
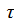
-*∆θ* curves and wrinkling characteristics as plotted in the figure below. Our recorded
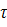
-∆*θ* curve shows a peak value of 531*nN*nm* as well as three turning points at ∆*θ*_1_=3°, ∆*θ*_2_=11°, ∆*θ*_3_=17°, which corresponds to the moment of wrinkle initiation, failure initiation, complete failure. The results are consistent with the reported peak value 591*nN*nm* and ∆*θ*_1_=4°,∆*θ*_2_=9°,∆*θ*_3_=15° by Qin *et al.*

**
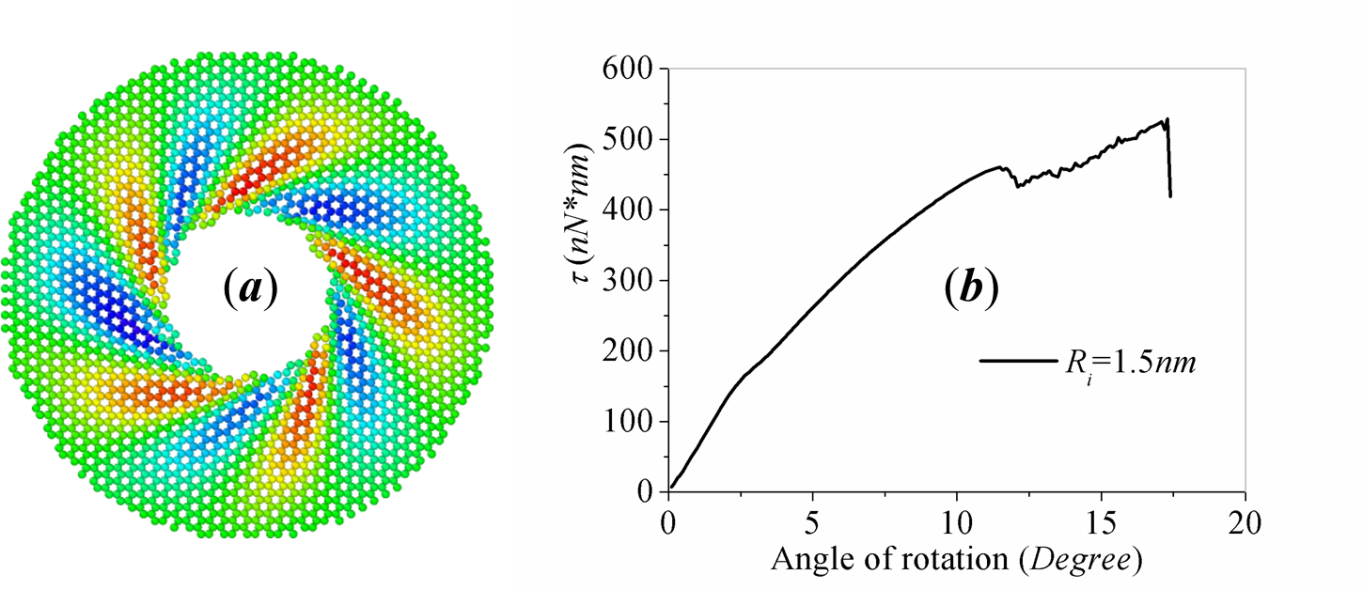
**

**Figure S4 |** Circular graphene annulus under in-plane rotation at inner edge with *R_i_*=1.5*nm*, *R_o_*=4.5*nm*. (***a***) The evolution of torque with outer boundary rotation angle. (***b***) Wrinkle amplitude contours of graphane annulus by coloring each atom according to the out-of-plane displacement.

Furthermore, we applied out models for the wrinkle characteristics of circular graphene annulus with *R_i_*=3*nm*, *R_o_*=9*nm* at 1K which has been studied by Tian *et al* [2]. The evolution of wrinkle amplitude with rotational angle is recorded as below. During the dynamic rotational process, the wrinkle amplitude grows from 0.8nm to 0.27nm which is consistent with the results reported by Tian *et al* (amplitude increases from 0.08*nm* to 0.26*nm*), The wrinkle profile at critical rotational angle shown in Fig. 2.1*b* has the same wrinkle number as the results reported by Tian *et al*.

**
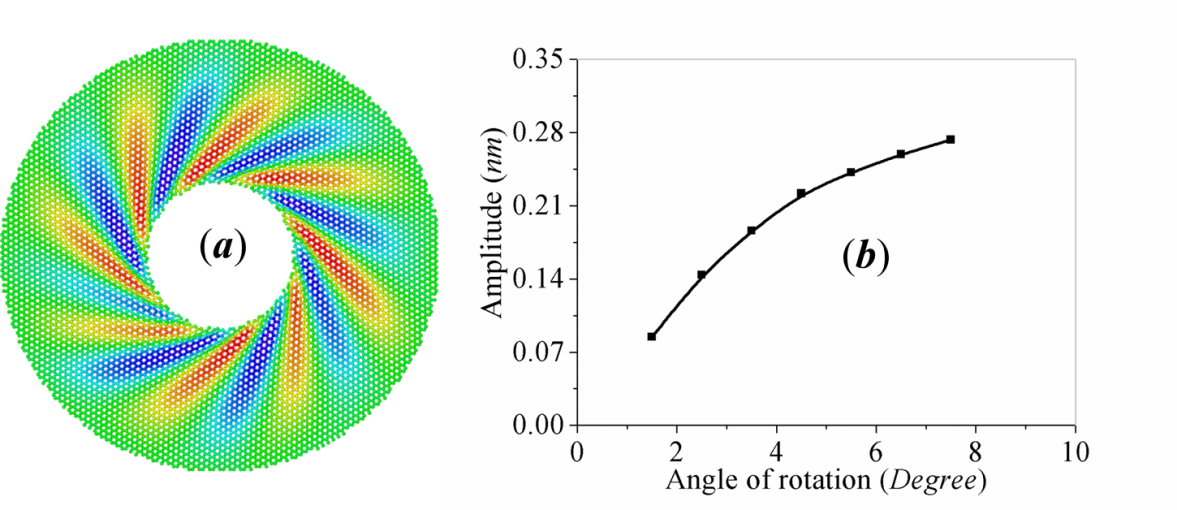
**

**Figure S5 | Circular graphene annulus under in-plane rotation at inner edge with *R_i_*=3*nm*, *R_o_*=9*nm* at 1*K*.** (***a***) Wrinkle amplitude contours of graphane annulus by coloring each atom according to the out-of-plane displacement. (***b***) The evolution of wrinkle amplitude with outer boundary radius *R_o_*.

What’s more, the wrinkle profile discussed in Fig. 1*b* of the main text shows *a* spiral angle of *δ*=19° which also agrees well with the value 20° reported by Wang *et al* [3] and the value 18° reported by Zhang *et al* [4]. These agreements confirm that our MD simulations are appropriate and reliable. Our recent work on the torque capacity of surface functionalized graphene annulus also adopts the same simulation procedures.[5]

The advancement of fabrication technology permits graphene nanosheets to be tailored into designed shapes for nano-devices. Since circular structures only accounts for a small proportion in engineering structures while structures with shapes of elliptical, square, [triangle](javascript:void(0);), and rectangle widely exist in engineering structures, circuits and electrical devices. To better prepare annular graphene for practical application, we constructed annulus with varying outer boundary for the shape effect of outer boundary on torque capacity of graphene annulus. Fig. S6*a*, *b* shows the atomistic structure of annulus with square and triangular outer boundaries. Similar to circular graphene annulus, square and [equilateral](javascript:void(0);) triangular annuli are featured by two parameters, inner radius *R_i_* and the radius of inscribed circle, i.e., outer radius *R_o_*. The structures shown in Fig. S6*a* have same radius *R_i_*=3*nm*, *R_o_*=8*nm*. By repeating the simulation described in Fig. 1*f* of the main text, we can get the *τ*-*∆θ* curves and the torque capacity for annulus with different outer boundary under circular rotation at inner edge. Fig. S6*c*-*f* shows the change of torque and wave number for square and [equilateral](javascript:void(0);) triangular graphene annulus with fixed *R_i_* =3*nm* and *R_o_* ranging from 4-10*nm*. With the increase of outer radius *R_o_*, the torque capacity remains approximate to 1750*nN*nm* while the outer radius and the wave number decreases sharply with *R_o_* and reach constant when *R_o_* is large enough for both of the two equilateral polygon models. The wrinkle amplitude and spiral angle are not given because their value is non-uniform at different areas and hard to count owning to the effect of outer boundary. These variation trends can also be observed in circular annulus, meaning the effect of equilateral polygon boundary on the mechanical properties of graphene annulus under in-plane circular shearing can be revealed effectively by studying the annulus with circular outer boundary.


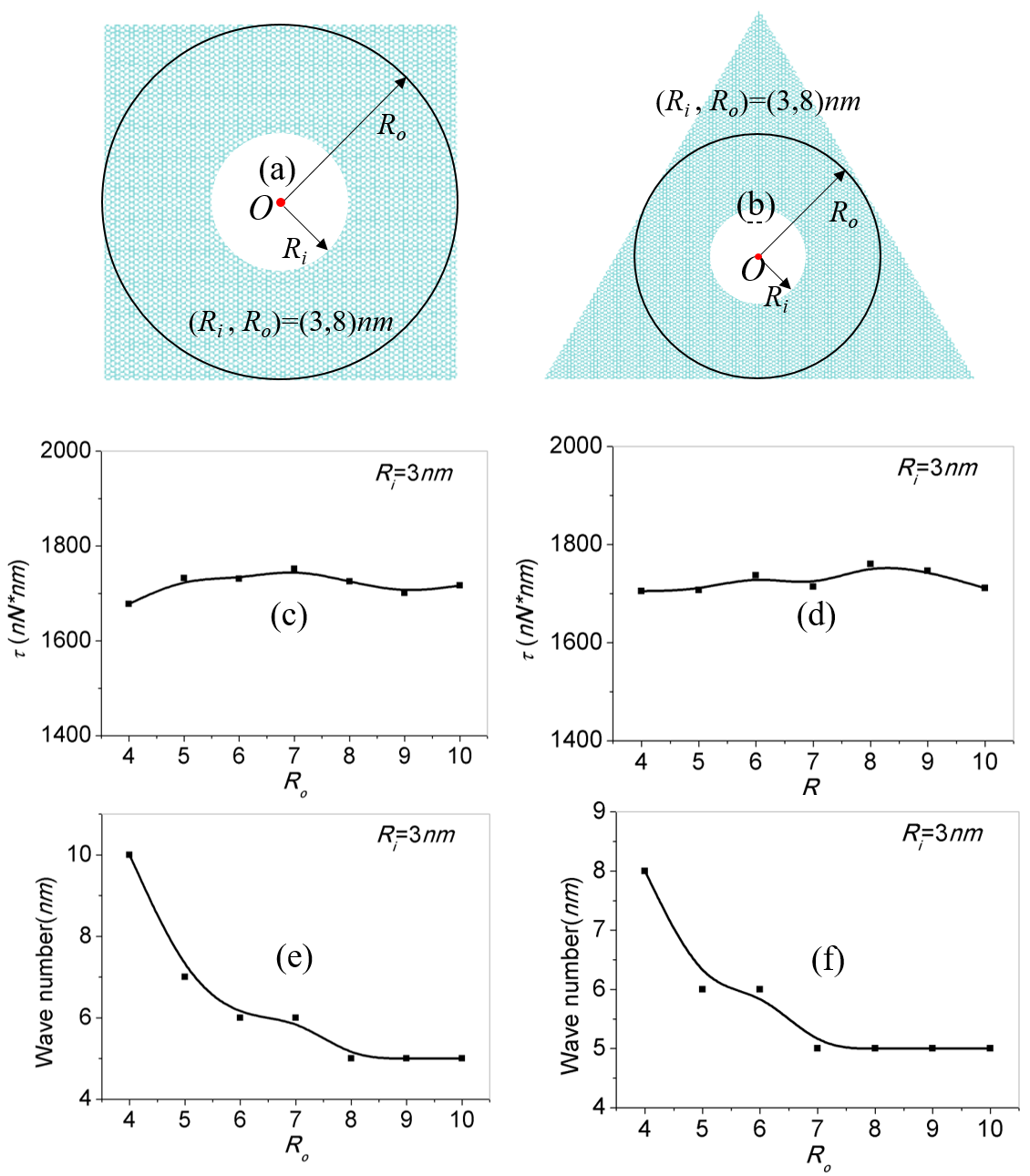


**Figure S6 | Shape effects of outer boundary on the torque capacity of graphene annulus with circular inner radius.** (***a*-*b***) Atomistic structure of square and triangular annulus with featured radius (*R_i_*, *R_o_*)=(3, 8)*nm*; (***c*-*d***) Evolution of torque capacity with the increase of outer radius *R_o_* for annulus with fixed *R_i_*. (***e*-*f***) Evolution of wave number with the increase of outer radius *R_o_* for annulus with fixed *R_i_*.

In order to explain the mechanism for the coupled effect of boundary aspect ratio on torque capacity, we further compared the torque capacity of graphene annulus with elliptical and rectangular outer boundaries. The outer boundary of elliptical and rectangular annulus can be described with feature parameters, minor-axis radius *R_os_* and major-axis radius *R_ol_*. By calculating the evolution of torque capacity with the increase of major axis radius *R_ol_* for annulus with fixed *R_i_*, *R_os_*, we noticed that the results of annulus with rectangular outer boundary is constant with the results elliptical annulus with same featured radius (Fig. S7). Thus, we choose elliptical annulus, whose wrinkle features and stress distribution can be described more conveniently, as a typical geometry for the effects of boundary aspect ratio and radii ratio *R_os_*/*R_i_* on torque capacity.


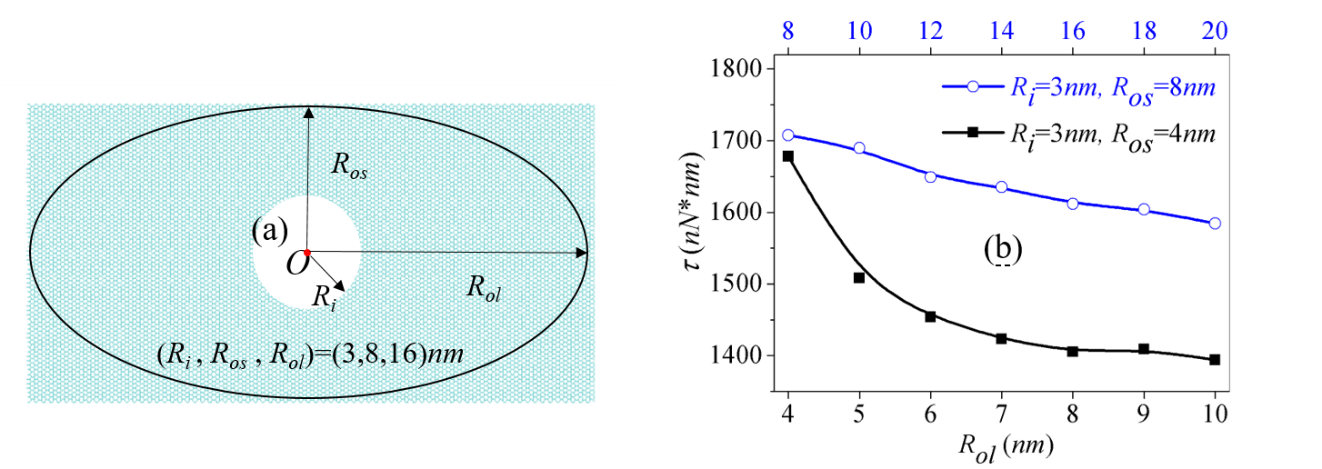

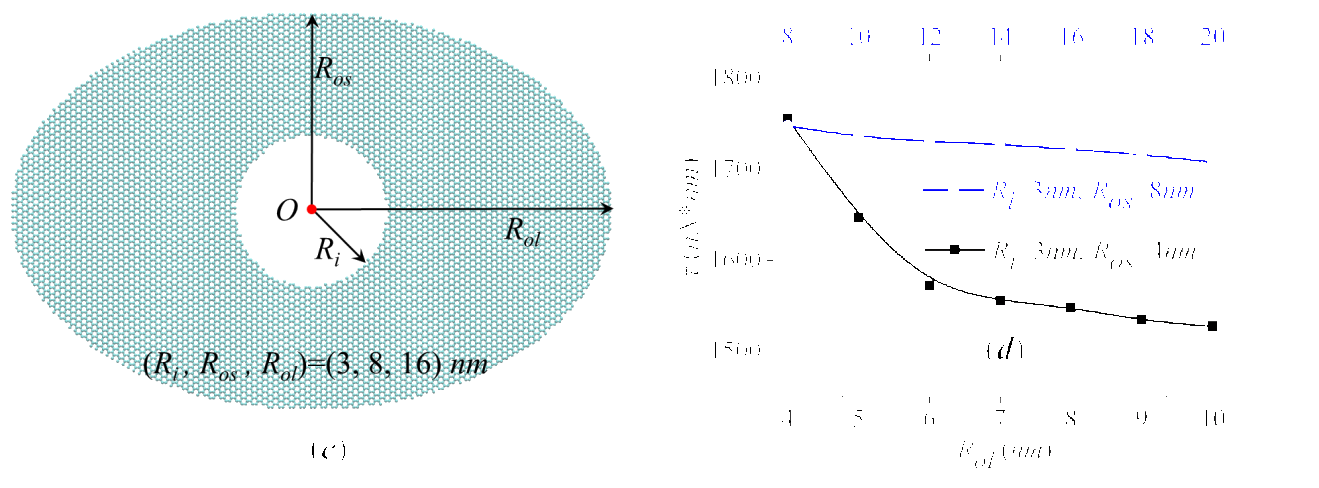


**Figure S7 | Effect of outer boundary aspect ratio on the torque capacity of graphene annulus with rectangular and elliptical outer boundary.** (***a***) Atomistic structure of rectangular annulus with (*R_i_*, *R_os_*, *R_ol_*)=(3, 8, 16)*nm* (***b***) Evolution of torque capacity with the increase of major axis radius *R_ol_* for annulus with fixed *R_i_*, *R_os_*. (***c***) Atomistic structure of elliptical annulus with (*R_i_*, *R_os_*, *R_ol_*)=(3, 8, 16)*nm* (***b***) Evolution of torque capacity with the increase of major axis radius *R_ol_* for annulus with fixed *R_i_*, *R_os_*.

1. Qin, Z., Taylor, M., Hwang, M., Bertoldi, K. & Buehler, M.J. Effect of Wrinkles on the Surface Area of Graphene: Toward the Design of Nanoelectronics. *Nano Lett.* **14**, 6520-6525 (2014).

2. Tian, Y., Li, Z. & Cai, K. Wrinkling Behaviour of Annular Graphynes under Circular Shearing Load Using Molecular Dynamics Simulations. *Nanomaterials and Nanotechnology* **5** (2015).

3. Wang, J.-W., Cao, Y.-P. & Feng, X.-Q. Archimedean spiral wrinkles on a film-substrate system induced by torsion. *Appl. Phys. Lett.* **104**, 031910 (2014).

4. Zhang, Z., Duan, W.H. & Wang, C.M. Tunable wrinkling pattern in annular graphene under circular shearing at inner edge. *Nanoscale* **4**, 5077-5081 (2012).

5. Li, Y., Liu, S., Datta, D. & Li, Z. Surface hydrogenation regulated wrinkling and torque capability of hydrogenated graphene annulus under circular shearing. *Sci. Rep.* **5**, 16556 (2015).
