# Supplementary figures and images for: Boundary-dependent mechanical properties of graphene annular under in-plane circular shearing via atomistic simulations
Source: Sci Rep. 2017 Feb 13;7:41767. doi: 10.1038/srep41767 (PMC5304194; doi:10.1038/srep41767)

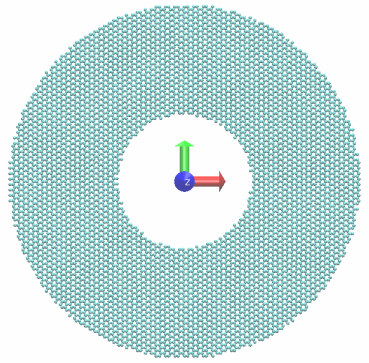

Supplement: Supplementary Video S1 [file srep41767-s2.gif]

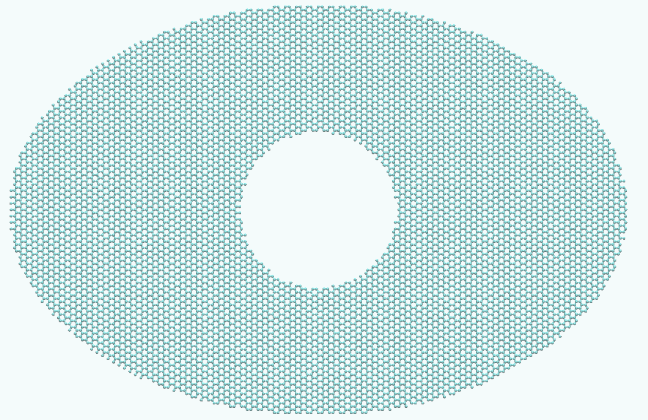

Supplement: Supplementary Video S2 [file srep41767-s3.gif]

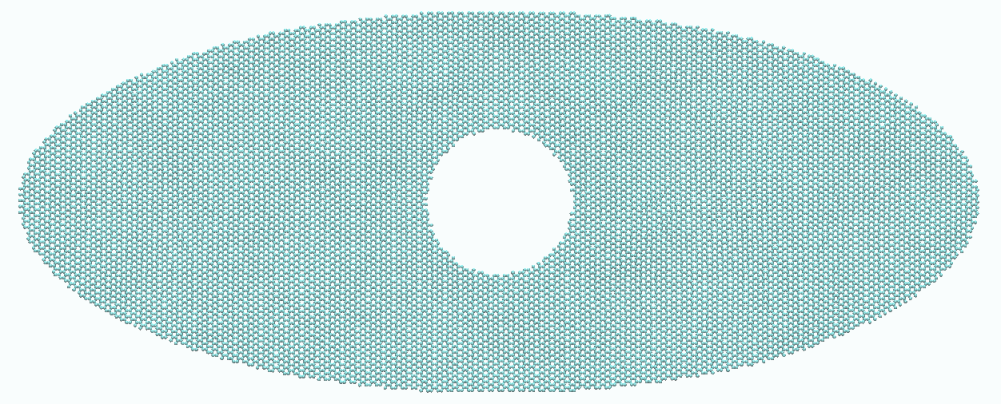

Supplement: Supplementary Video S3 [file srep41767-s4.gif]
